# Mechanistic in vitro studies indicate that the clinical drug–drug interactions between protease inhibitors and rosuvastatin are driven by inhibition of intestinal BCRP and hepatic OATP1B1 with minimal contribution from OATP1B3, NTCP and OAT3

**DOI:** 10.1002/prp2.1060

**Published:** 2023-02-22

**Authors:** Robert Elsby, Hannah Coghlan, Jacob Edgerton, David Hodgson, Samuel Outteridge, Hayley Atkinson

**Affiliations:** ^1^ Department of Drug Transporter Sciences, Cyprotex Discovery Ltd (an Evotec Company) Macclesfield Cheshire UK; ^2^ Present address: Department of Pharmacology and Therapeutics, MRC Centre for Drug Safety Science University of Liverpool Liverpool UK

## Abstract

Previous use of a mechanistic static model to accurately quantify the increased rosuvastatin exposure due to drug–drug interaction (DDI) with coadministered atazanavir underpredicted the magnitude of area under the plasma concentration–time curve ratio (AUCR) based on inhibition of breast cancer resistance protein (BCRP) and organic anion transporting polypeptide (OATP) 1B1. To reconcile the disconnect between predicted and clinical AUCR, atazanavir and other protease inhibitors (darunavir, lopinavir and ritonavir) were evaluated as inhibitors of BCRP, OATP1B1, OATP1B3, sodium taurocholate cotransporting polypeptide (NTCP) and organic anion transporter (OAT) 3. None of the drugs inhibited OAT3, nor did darunavir and ritonavir inhibit OATP1B3 or NTCP. All drugs inhibited BCRP‐mediated estrone 3‐sulfate transport or OATP1B1‐mediated estradiol 17β‐D‐glucuronide transport with the same rank order of inhibitory potency (lopinavir>ritonavir>atazanavir>>darunavir) and mean IC_50_ values ranging from 15.5 ± 2.80 μM to 143 ± 14.7 μM or 0.220 ± 0.0655 μM to 9.53 ± 2.50 μM, respectively. Atazanavir and lopinavir also inhibited OATP1B3‐ or NTCP‐mediated transport with a mean IC_50_ of 1.86 ± 0.500 μM or 65.6 ± 10.7 μM and 5.04 ± 0.0950 μM or 20.3 ± 2.13 μM, respectively. Following integration of a combined hepatic transport component into the previous mechanistic static model using the in vitro inhibitory kinetic parameters determined above for atazanavir, the newly predicted rosuvastatin AUCR reconciled with the clinically observed AUCR confirming additional minor involvement of OATP1B3 and NTCP inhibition in its DDI. The predictions for the other protease inhibitors confirmed inhibition of intestinal BCRP and hepatic OATP1B1 as the principal pathways involved in their clinical DDI with rosuvastatin.

## Graphical abstract

This work sought to clarify the mechanisms underlying clinical DDIs between rosuvastatin and protease inhibitors. Investigating in vitro inhibition of the critical transporter disposition pathways of rosuvastatin, and subsequent incorporation of cell test system derived determined K_i_ values into mechanistic static equations, enabled successful quantitative prediction of clinical AUCRs. Predictions suggest the mechanisms driving DDI are principally inhibition of intestinal BCRP and hepatic OATP1B1, with minimal contribution from OATP1B3, NTCP or OAT3.

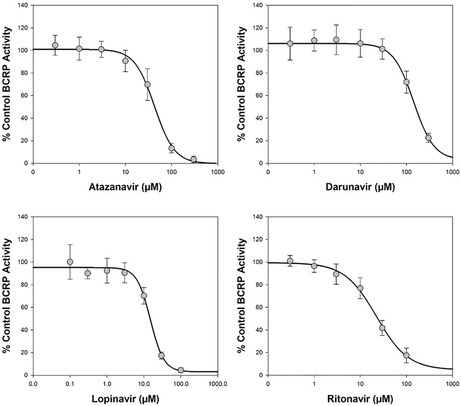

**Abbreviations**
AUC: area under the plasma concentration–time curve
AUCR: area under the plasma concentration–time curve ratio
BCRP: breast cancer resistance protein
C_max_
: maximum plasma concentration
DDI: drug–drug interaction
DMSO: dimethyl sulfoxide
DPM: disintegrations per minute
*ƒ*_e_
: fractio n excreted value
HEK293: human embryonic kidney 293
IC_50_
: concentration that produces 50% inhibition
OAT: organic anion transporter
OATP: organic anion transporting polypeptide
NTCP: sodium taurocholate cotransporting polypeptide

## 1 INTRODUCTION

Statins are widely prescribed for the effective treatment of hypercholesterolemia and as a drug class are generally well tolerated in humans.^1^ However, in up to 29% of patients, adverse effects associated with myopathy have been reported with their use, ranging from mild muscle pain to (in extreme cases) fatal rhabdomyolysis.^2^ Because of this it is imperative that careful consideration be given to factors that may have an impact on the disposition and therefore exposure of statins. Consequently, drug–drug interactions (DDIs) with statins are of clinical concern as elevated plasma concentrations of statins, due to inhibition of critical disposition pathways by a co‐medication, are associated with increased muscle exposure and therefore risk of myopathy.^3^ Statins are increasingly being prescribed in human immunodeficiency virus (HIV)‐infected patients since dyslipidemias are common comorbidities of HIV infection,^4^ as identified in 24% of patients.^5^ As a result of their subsequent widespread use in HIV patients,^6^ it should not come as a surprise that clinically significant DDIs resulting in myotoxicities have been observed between antiretroviral protease inhibitors and statin drugs.^7^

A particular concern is the potential for DDI between protease inhibitors and the hydrophilic statin rosuvastatin, which has been shown to be more effective in managing dyslipidemia in HIV patients than other statins and has one of the largest prescription frequencies amongst the statins.^6^, ^8^ In fact, clinically significant DDIs resulting in up to 3‐fold increases in rosuvastatin exposure (defined as area under the plasma concentration–time curve, AUC) have been observed between rosuvastatin and the perpetrators atazanavir, darunavir or lopinavir/ritonavir (Crestor® drug label)^9^, ^10^, ^11^, ^12^. The critical disposition pathways of rosuvastatin and their contributions to overall clearance (fraction excreted [*ƒ*_e_] values) have been determined previously.^1^ These critical pathways include intestinal breast cancer resistance protein (BCRP) efflux as the barrier to absorption (fraction absorbed = 0.5, therefore BCRP *ƒ*_e_ = 0.5), hepatic organic anion transporting polypeptide 1B1 (OATP1B1, *ƒ*_e_ = 0.38) uptake for hepatic elimination, and active renal secretion of rosuvastatin mediated by organic anion transporter 3 (OAT3, *ƒ*_e_ = 0.25) uptake. Other minor pathways that contribute toward the overall hepatic elimination of rosuvastatin (*ƒ*_e_ = 0.7) include OATP1B3 (*ƒ*_e_ = 0.11) and sodium taurocholate cotransporting polypeptide (NTCP, *ƒ*_e_ = 0.21).^13^ Although the mechanisms underlying rosuvastatin‐protease inhibitor DDIs are not yet fully characterized, studies by Annaert et al.,^14^ Weiss et al.^15^ and Elsby et al.^13^ established that many protease inhibitors are in vitro inhibitors of the key disposition transporters BCRP and OATP1B1. Moreover, Elsby et al.^13^ used their published mechanistic static model to predict accurately the AUC ratio (AUCR) of rosuvastatin due to inhibition of BCRP and OATP1B1 pathways by darunavir and lopinavir, establishing that these specific DDIs are primarily driven by perturbation of BCRP function, and to a lesser extent by OATP1B1. However, for atazanavir, inhibition of BCRP and OATP1B1 alone could not fully reconcile its clinically observed DDI with rosuvastatin (predicted AUCR = 2.13 versus clinical AUCR = 3.1). Only when the overall hepatic uptake pathway (*ƒ*_e_ = 0.7) was considered using a literature OATP1B1 K_i_ as presumed surrogate for inhibition of “overall active hepatic uptake”, did the mechanistic approach predict (AUCR = 3.25) the clinical observation.^16^ The assumptions made for that prediction were that atazanavir is indeed an inhibitor of minor OATP1B3 and NTCP, with K_i_ values similar to the surrogate OATP1B1 value, and that inhibition of renal OAT3 does not contribute to DDI.

Therefore, in order to test this hypothesis, the aims of study were to determine the inhibitory parameters (IC_50_) of atazanavir versus BCRP and OATP1B1 and to investigate atazanavir as an inhibitor of the minor rosuvastatin disposition pathway transporters OATP1B3, NTCP and OAT3 in vitro. Using these data in the mechanistic static rosuvastatin model described by Elsby et al.^13^ now integrating the combined individual hepatic transporter components for OATP1B1, OATP1B3 and NTCP for “overall active hepatic uptake”, an attempt was made to predict the clinical AUCR for rosuvastatin in order to confirm the underlying mechanism behind the DDI perpetrated by atazanavir. For completeness to define fully the mechanisms underlying the other rosuvastatin‐protease inhibitor drug DDIs, the final aim of the study was to perform the same in vitro transporter assessments and mechanistic predictions above for the remaining clinically important perpetrator drugs darunavir, lopinavir and ritonavir.

## 2 MATERIALS AND METHODS

### 2.1 Materials

Estrone 3‐sulfate sodium salt, estradiol 17β‐D‐glucuronide sodium salt, sodium taurocholate hydrate, novobiocin, rifamycin SV sodium salt, cyclosporin A, pioglitazone hydrochloride, probenecid, lucifer yellow dilithium salt, sodium butyrate, non‐essential amino acids and HEPES were purchased from Sigma‐Aldrich (Poole, Dorset, UK). [^3^H]‐Estrone 3‐sulfate, [^3^H]‐estradiol 17β‐D‐glucuronide, [^3^H]‐taurocholic acid, Ultima Gold XR liquid scintillation cocktail and liquid scintillation counting 24‐well visiplates or 96‐well isoplates were purchased from PerkinElmer Life and Analytical Sciences (Buckinghamshire, UK). Hanks balanced salt solution (Gibco™ HBSS; containing CaCl_2_ and MgCl_2_), Dulbecco's modified eagle medium (Gibco™ DMEM: high glucose with GlutaMax and pyruvate for human embryonic kidney (HEK)293 transfected cells; Gibco™ DMEM: high glucose and pyruvate for Caco‐2 cells), fetal bovine serum (Gibco™: non‐heat inactivated for HEK293 transfected cells; heat inactivated for Caco‐2 cells), mammalian protein extraction reagent (M‐PER), bicinchoninic acid protein assay kit, and 45% glucose solution were purchased from Fisher Scientific (Loughborough, UK). All other chemicals, solvents and reagents were purchased from Fisher Scientific.

Biocoat™ Poly‐D‐lysine 24‐well multiwell plates, human organic anion transporting polypeptide (OATP) 1B1 (SLCO1B1)‐, human OATP1B3 (SLCO1B3)‐, human sodium taurocholate cotransporting polypeptide (NTCP; SLC10A1)‐, and human organic anion transporter (OAT) 3 (SLC22A8)‐expressing HEK293 TransportoCells™ and vector control cells were supplied by Corning BV Life Sciences (Amsterdam, The Netherlands). Millicell‐96 multiwell cell culture insert plates (with polycarbonate membranes; 0.4 μm pore size, 0.11 cm^2^ surface area) and Millicell 96‐well transport analysis plates were purchased from Millipore (Watford, Hertfordshire, UK). Caco‐2 cells were obtained from American Type Culture Collection. 24‐Well tissue culture treated microplates and various 96‐well v‐bottom or round‐bottomed polypropylene microplates were supplied by Fisher Scientific.

### 2.2 Assessment of BCRP inhibition (IC_50_ determination)

The methodology used was adapted from the previously validated Caco‐2 unidirectional (basolateral to apical; B‐A) BCRP inhibition assay.^13^ Briefly, Caco‐2 cells between passage numbers 40‐60 were seeded onto Millicell‐96 multiwell cell culture insert plates at 1 × 10^5^ cells/cm^2^ and cultured at 37°C in an atmosphere of 5 % CO_2_ with a relative humidity of 95%. The cells were cultured in medium (consisting of DMEM supplemented with 10% (w/v) fetal bovine serum, 2 mM L‐glutamine, 1% (v/v) nonessential amino acids and 50 U mL^−1^ penicillin and 50 μg mL^−1^ streptomycin) which was changed every two or three days.

The BCRP inhibition experiments were performed between day 18 to 22 post‐seeding. The cell monolayers were washed twice with pre‐warmed (37°C) transport buffer (HBSS containing 25 mM HEPES and 4.45 mM glucose, pH 7.4) before being pre‐incubated with transport buffer containing protease inhibitor drug or positive control inhibitor (novobiocin) on both sides of the cell monolayer for 30 min at 37°C. After pre‐incubation, transport buffer was removed and the appropriate donor solutions were added to the basolateral donor compartment. Donor solutions (final DMSO concentration of ≤1 % v/v) in transport buffer contained the probe substrate [^3^H]‐estrone 3‐sulfate (1 μM; approximately 7‐fold lower than K_m_ for BCRP = 7.4 μM)^17^ and either DMSO (vehicle control), protease inhibitor drug (atazanavir, darunavir or ritonavir at 0.3, 1, 3, 10, 30, 100 and 300 μM; lopinavir at 0.1, 0.3, 1, 3, 10, 30 and 100 μM), or positive control inhibitor (novobiocin; 0.1, 0.3, 1, 3, 10, 30 and 100 μM) and were added to the basolateral compartments (total volume = 210 μL). Receiver solutions (final DMSO concentration of ≤1 % v/v) in transport buffer containing either the corresponding concentration of test inhibitor or DMSO (vehicle control), and the fluorescent cell monolayer integrity marker lucifer yellow (100 μM), were added to the apical compartments (total volume = 90 μL). Incubations were conducted in triplicate wells per assay condition in a heated incubator at 37°C for 90 min. Following the incubation, the amount (pmol) of [^3^H]‐estrone 3‐sulfate appearing in the receiver compartment was determined by sampling (50 μL) into a white walled clear bottomed multiwell plate, to which scintillation fluid (200 μL) was added, and then samples counted on a Microbeta2 liquid scintillation counter (PerkinElmer). The quantified amount (derived from disintegrations per minute; DPM) of [^3^H]‐estrone 3‐sulfate was subsequently converted to concentration (pmol/mL) and used to calculate apparent permeability (P_app_), as described previously.^18^ The passive permeability of estrone 3‐sulfate observed when BCRP is completely inhibited (derived from incubations containing the highest concentration (100 μM) of the positive control inhibitor novobiocin) was subtracted from the determined B‐A P_app_ value in the absence or presence of test inhibitor, to give a corrected BCRP‐mediated B‐A P_app_, which was subsequently converted to percentage (vehicle) control transport activity. For each test inhibitor, determined percentage control transport activity was plotted against nominal inhibitor concentration and fitted using SigmaPlot 12.5 (Systat Software Inc., Chicago, IL; four parameter logistic equation) to determine the concentration that produces half‐maximal inhibition of probe substrate transport (IC_50_; equivalent to K_i_ as probe substrate concentration in the assay is at least 7‐times lower than its K_m_, and assuming competitive inhibition). The P_app_ of co‐incubated lucifer yellow across the cell monolayer was determined and cell monolayer integrity was deemed acceptable if P_app_ < 1.0 × 10^−6^ cm/s.

### 2.3 Assessment of SLC transporter inhibition (single concentration screen)

OATP1B3, NTCP, OAT3 and vector control cell lines were seeded in cell culture medium (consisting of DMEM supplemented with 10% (w/v) fetal bovine serum and 1% (v/v) nonessential amino acids) into 24‐well poly‐D‐lysine coated plates at 3‐4 × 10^5^ cells per well to achieve a pre‐assay confluence of typically 80%–95%. Cells were cultured at 37°C, 8% CO_2_ for 24 h and media was replaced 3–4 h post‐seeding with either fresh media for OAT3 cells (and corresponding control cells) or fresh media containing 2 mM sodium butyrate for all other cell lines. Prior to assay, cells were washed twice with pre‐warmed uptake buffer (HBSS containing 10 mM HEPES, pH 7.4) then pre‐incubated with test inhibitor drug (at the single concentration stated below) at 37°C in warm uptake buffer for 15 min. After the pre‐incubation step, uptake buffer was removed and the appropriate corresponding incubation solutions were added to the wells.

Uptake of probe substrate (OATP1B3: [^3^H]‐estradiol 17β‐D‐glucuronide, 0.02 μM; NTCP: [^3^H]‐taurocholic acid, 1 μM; OAT3: [^3^H]‐estrone 3‐sulfate, 1 μM) was determined (in triplicate wells per condition, on two separate occasions) over a specified linear incubation time (OATP1B3 = 2 min; NTCP and OAT3 = 3 min) at 37°C in SLC transporter‐expressing cells and vector control cells, in the absence and presence of protease inhibitor drug at a single tested concentration, or positive control inhibitor (OATP1B3: 10 μM cyclosporin A; NTCP: 50 μM pioglitazone; OAT3: 300 μM probenecid). The final dimethyl sulfoxide (DMSO) concentration was 0.1% (v/v). The chosen concentration of each drug represented ≥10x its unbound maximum hepatic inlet concentration for hepatic OATP1B3 and NTCP, or ≥10x its unbound maximum plasma concentration for renal OAT3. This translated to tested concentrations of 50 and 15 μM for atazanavir, 15 and 10 μM for darunavir, 5 and 3 μM for lopinavir, or 0.3 and 0.1 μM for ritonavir against OATP1B3/NTCP and OAT3, respectively. Following the incubation period, active transport processes were stopped by removing (via aspiration) the incubation solutions and immediately washing the cells twice with ice cold uptake buffer before then placing plates on ice. Following the wash steps, M‐PER (400 μL) was added to each well and cells were lysed for at least 5 min at 250 rpm on an orbital shaker. An aliquot (300 μL) of cell lysate was added to a white walled, clear bottomed 24‐well visiplate, liquid scintillation cocktail (2 mL) was added, and samples were counted on a Microbeta2 scintillation counter in order to determine the total radioactivity (DPM) taken up in cells. In parallel, the protein content of cell lysates (25 μL) was determined using a Bicinchoninic acid (BCA) protein assay kit according to the manufacturer's instructions.

For data analysis, the determined total uptake of radiolabeled probe substrate into cells (pmol) was normalized to the protein (mg) content of each well to calculate the uptake activity (pmol/mg). Uptake activity of probe substrate into vector control cells was subtracted from that determined into transporter‐expressing cells to calculate the corrected transporter‐mediated uptake. Corrected uptake activity (pmol/mg) was subsequently converted to percentage (vehicle) control transport activity.

### 2.4 Assessment of SLC transporter inhibition (IC_50_ determination)

OATP1B1, OATP1B3, NTCP and vector control cell lines were cultured and subsequent transporter assays conducted as described above using the same transporter‐specific probe substrates and incubation times. For OATP1B1, the probe substrate was [^3^H]‐estradiol 17β‐D‐glucuronide (0.02 μM) incubated for 2 min, with rifamycin SV (100 μM) as positive control inhibitor. For IC_50_ determinations, the only difference to the method performed above was that the 15‐min pre‐incubation step contained a range of six concentrations of protease inhibitor drug, and subsequent incubations were conducted with the same six concentration levels of drug rather than with a single concentration. All four protease inhibitor drugs were studied against OATP1B1 using pre‐incubation/incubation concentrations of either 0.1, 0.3, 1, 3, 10 and 30 μM for atazanavir and darunavir, or 0.03, 0.1, 0.3, 1, 3 and 10 μM for lopinavir and ritonavir. Based upon the results determined from the inhibition screen, only atazanavir and lopinavir were studied against OATP1B3 or NTCP using concentrations of 0.1, 0.3, 1, 3, 10 and 30 μM and 0.03, 0.1, 0.3, 1, 3 and 10 μM, or 0.3, 1, 3, 10, 30 and 50 μM and 0.1, 0.3, 1, 3, 10 and 20 μM, respectively.

For each drug, determined percentage (vehicle) control transport activity was plotted against nominal inhibitor concentration and fitted using SigmaPlot 12.5 (Systat Software Inc., Chicago, IL; four parameter logistic equation) to determine the concentration that produces half‐maximal inhibition of probe substrate transport (IC_50_; equivalent to K_i_ assuming competitive inhibition as probe substrate concentration in the assay is at least 10‐times lower than its K_m_).

### 2.5 Mechanistic static prediction of rosuvastatin AUC change for the known clinical DDIs perpetrated by protease inhibitors based upon determined in vitro BCRP, OATP1B1, OATP1B3, NTCP and OAT3 inhibitory parameters

The mean IC_50_ (equating to K_i_) values obtained for atazanavir, darunavir, lopinavir and ritonavir versus a range of transporters were incorporated into the adapted Rowland‐Matin mechanistic static equation (Equation 1) previously described by Elsby et al.^1^, ^13^, ^19^ in order to predict the change in rosuvastatin AUC based upon inhibition of an BCRP, OATP1B1, OATP1B3, NTCP, all combined hepatic transporter (OATP1B1/OATP1B3/NTCP; Equation 2), and OAT3 fraction excreted (*ƒ*_e_) value of 0.5, 0.38, 0.11, 0.21, 0.7 and 0.25, respectively.^13^ Overall AUCR arising from multiple pathways, is simply the product (multiplication) of the AUCR determined for each separate ADME pathway, e.g., for combined inhibition of intestinal BCRP and hepatic OATP1B1, the overall AUCR is equal to the Equation 1‐derived AUCR for BCRP × the Equation 1‐derived AUCR for OATP1B1. For inhibition of BCRP and of combined hepatic uptake as one process (e.g., OATP1B1+OATP1B3+NTCP), then it would be the Equation 1‐derived AUCR for BCRP × the Equation 2‐derived AUCR (combined OATP1B1+OATP1B3+NTCP).

(1) $$\text{AUCR}=\frac{1}{\frac{{ƒ}_{e}}{\left(1+\left[I\right]/K_{i}\right)}+\left(1-{ƒ}_{e}\right)\;}$$

(2) $$\begin{array}{c} \text{AUCR}=\frac{1}{\frac{{ƒ}_{e}\left(\text{Transporters}\;1+2+3\right)}{\left(1+\left(\left[I\right]/K_{i}\right)\text{Trspt}1+\left(\left[I\right]/K_{i}\right)\text{Trspt}2+\left(\left[I\right]/K_{i}\right)\text{Trspt}3\;\right)}+\left(1-{ƒ}_{e\left(\text{Transporters}\;1+2+3\right)}\right)\;} \end{array}$$

where K_i_ = absolute inhibition constant (equating to IC_50_ for transporters as the probe [S] <<<< K_m_ in the transporter inhibition assay and assuming competitive inhibition, based on the Cheng‐Prusoff equation)^20^ and [I] = unbound maximum hepatic inlet concentration (I_in max u_ = f_u_ × (C_max_ + (((F_a_F_g_ × k_a_ × dose (mol))/Q_h_))/R_B_)) for hepatic transporters, or [I] = maximum enterocyte concentration (I_g_ = (F_a_F_g_ × k_a_ × dose (mol))/Q_ent_) for intestinal transporters,^19^ or [I] = unbound maximum plasma concentration at steady state (C_max u_ = f_u_ × C_max_) for renal transporters.^19^ f_u_ = unbound fraction in plasma, C_max_ = maximum total plasma concentration of inhibitor at steady state, F_a_F_g_ = fraction of the dose absorbed after oral administration, k_a_ = absorption rate constant (min^−1^), Q_h_ = hepatic blood flow (1617 mL/min), R_B_ is the blood‐to‐plasma concentration ratio (default = 1.0) and Q_ent_ = enterocyte blood flow (300 mL/min).

### 2.6 Nomenclature of targets and ligands

Key protein targets and ligands in this article are hyperlinked to corresponding entries in http://www.guidetopharmacology.org, the common portal for data from the IUPHAR/BPS Guide to PHARMACOLOGY,^21^ and are permanently archived in the Concise Guide to PHARMACOLOGY 2021/22: Transporters.^22^

## 3 RESULTS

### 3.1 Assessment of protease inhibitors as inhibitors of BCRP in vitro

**FIGURE 1:**
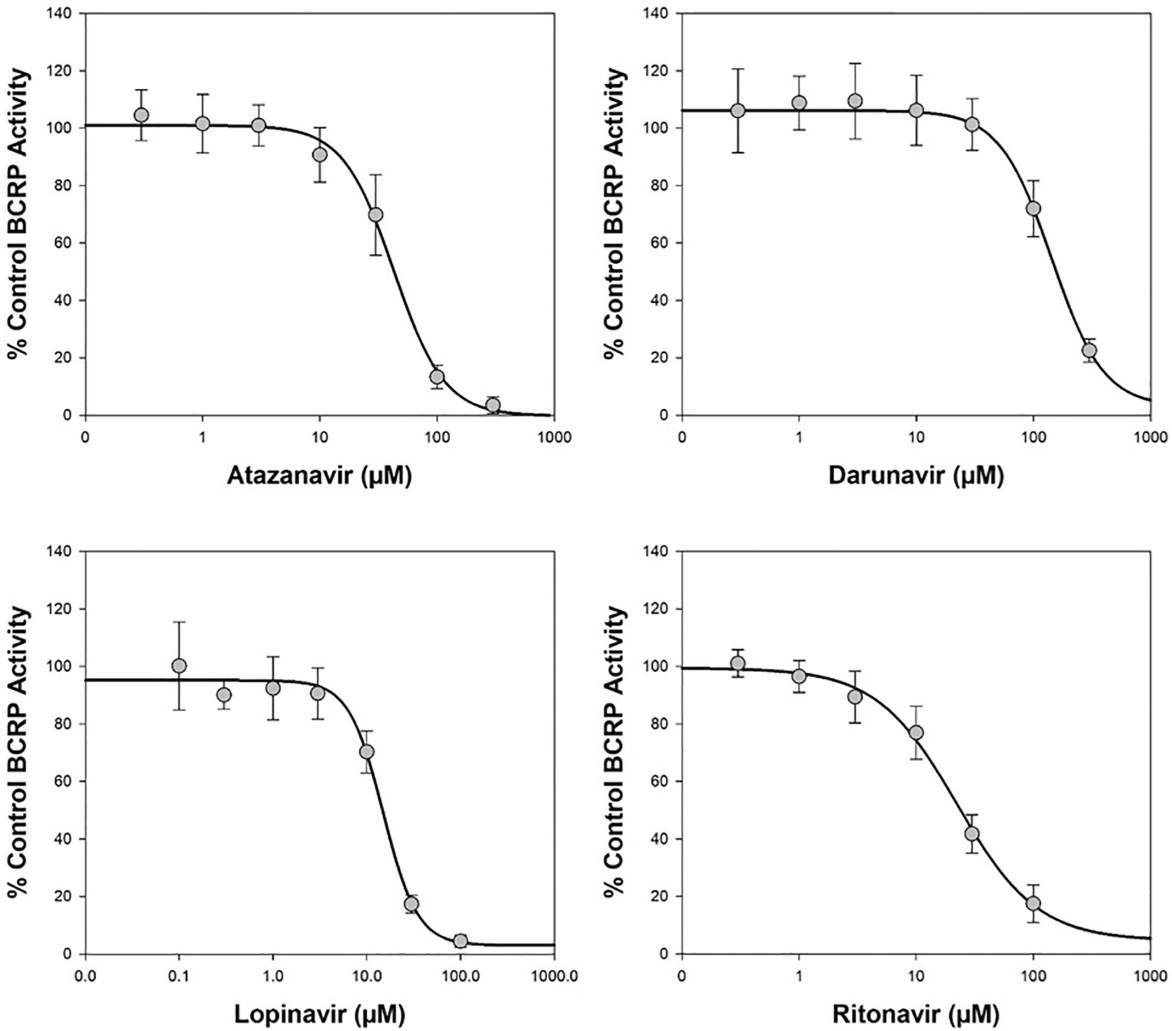
Representative plot of mean concentration‐dependent inhibition of BCRP‐mediated transport of [^3^H]‐estrone 3‐sulfate (1 μM) by atazanavir, darunavir, lopinavir or ritonavir. Data are expressed as mean (± standard deviation) from 12 wells per incubation condition (triplicate wells on four separate occasions) for atazanavir and ritonavir, or 9 wells per incubation condition (triplicate wells on three separate occasions) for darunavir and lopinavir.

Atazanavir, darunavir, lopinavir or ritonavir demonstrated reproducible inter‐assay concentration‐dependent inhibition of BCRP‐mediated transport of [^3^H]‐estrone 3‐sulfate (1 μM) with a determined mean (±SD) IC_50_ value of 42.2 ± 10.2 μM (Table 1 and Figure 1), 143 ± 14.7 μM (Table 2 and Figure 1), 15.5 ± 2.80 μM (Table 3 and Figure 1), or 25.4 ± 9.26 μM (Table 4 and Figure 1), respectively. Assessment of DDI potential via inhibition of intestinal BCRP using the current FDA^23^ static equation approach gave an [I_gut_] (dose (mol)/250 mL)/K_i_ value of 40 (1702/42.2), 31 (4382/143), 164 (2545/15.5) or 22 (555/25.4), respectively, indicating the potential for interaction in vivo (≥10) perpetrated by all drugs. The positive control inhibitor, novobiocin (0.1–100 μM) gave acceptable inhibition (mean IC_50_ of 1.81 ± 0.276 μM) in the test system.

**TABLE 1:** Determined in vitro K_i_ (IC_50_) values for inhibition of BCRP, OATP1B1, OATP1B3 and NTCP by atazanavir.

Transporter	IC_50_ (μM)								Published IC_50_/K_i_ (μM) (Probe substrate)	Reference
	Determined values from different occasions				Mean	SD	CV(%)	95% CI^a^		
BCRP	49.8	30.3	51.5	37.1	42.2	10.2	24.2	26.0–58.4	69.1 (pheophorbide A)	Weiss et al.^15^
OATP1B1	0.583	0.928		0.690	0.734	0.177	24.1	0.294–1.17	0.91 (atorvastatin)	Karlgren et al.^31^
OATP1B3	1.75	2.41		1.43	1.86	0.500	26.9	0.620–3.10	7.3 (atorvastatin)	Vildhede et al.^33^
NTCP	55.9	63.8		77.1	65.6	10.7	16.3	39.0–92.2	‐	Vildhede et al.^33^

*Note*: K_i_ = absolute inhibition constant versus the transporter (assuming competitive inhibition, this equates to IC_50_ in the assays as probe substrate concentration utilized is <<<< K_m_).

^a^ 95% confidence interval was calculated by adding or subtracting the product of the t‐distribution value (3.182 *n* = 4; 4.303 *n* = 3) and the value of the standard deviation divided by the square root of the sample size from the determined mean value.

**TABLE 2:** Determined in vitro K_i_ (IC_50_) values for inhibition of BCRP and OATP1B1 by darunavir.

Transporter	IC_50_ (μM)							Published IC_50_/K_i_ (μM) (Probe substrate)	References
	Determined values from different occasions			Mean	SD	CV(%)	95% CI^a^		
BCRP	126	154	148	143	14.7	10.3	107–180	75 (rosuvastatin)	Elsby et al.^13^
OATP1B1	12.4	7.89	8.29	9.53	2.50	26.2	3.32–15.7	4.3 (estradiol 17β‐D‐glucuronide)	Elsby et al.^13^

*Note*: K_i_ = absolute inhibition constant versus the transporter (assuming competitive inhibition, this equates to IC_50_ in the assays as probe substrate concentration utilized is <<<< K_m_).

^a^ 95% confidence interval was calculated by adding or subtracting the product of the t‐distribution value (4.303) and the value of the standard deviation divided by the square root of the sample size from the determined mean value.

**TABLE 3:** Determined in vitro K_i_ (IC_50_) values for inhibition of BCRP, OATP1B1, OATP1B3 and NTCP by lopinavir.

Transporter	IC_50_ (μM)							Published IC_50_/K_i_ (μM) (Probe substrate)	References
	Determined Values from Different Occasions			Mean	SD	CV(%)	95% CI^a^		
BCRP	12.7	18.3	15.6	15.5	2.80	18.1	8.54–22.5	8.7 (rosuvastatin)	Elsby et al.^13^
OATP1B1	0.291	0.207	0.162	0.220	0.0655	29.8	0.0570–0.383	0.43 (estradiol 17β‐D‐glucuronide)	Elsby et al.^13^
OATP1B3	5.14	5.04	4.95	5.04	0.0950	1.9	4.80–5.28	‐	Vildhede et al.^33^
NTCP	22.6	18.4	19.9	20.3	2.13	10.5	15.0–25.6	‐	Vildhede et al.^33^

*Note*: K_i_ = absolute inhibition constant versus the transporter (assuming competitive inhibition, this equates to IC_50_ in the assays as probe substrate concentration utilized is <<<< K_m_).

^a^ 95% confidence interval was calculated by adding or subtracting the product of the t‐distribution value (4.303) and the value of the standard deviation divided by the square root of the sample size from the determined mean value.

**TABLE 4:** Determined in vitro K_i_ (IC_50_) values for inhibition of BCRP and OATP1B1 by ritonavir

Transporter	IC_50_ (μM)								Published IC_50_/K_i_ (μM) (Probe substrate)	Reference
	Determined Values from Different Occasions				Mean	SD	CV(%)	95% CI^a^		
BCRP	38.6	22.1	17.1	23.6	25.4	9.26	36.5	10.7–40.1	19.5 (mitoxantrone)	Gupta et al.^30^
OATP1B1	0.565	0.574	0.353		0.497	0.125	25.2	0.186–0.808		

*Note*: K_i_ = absolute inhibition constant versus the transporter (assuming competitive inhibition, this equates to IC_50_ in the assays as probe substrate concentration utilized is <<<< K_m_).

^a^ 95% confidence interval was calculated by adding or subtracting the product of the t‐distribution value (3.182 *n* = 4; 4.303 *n* = 3) and the value of the standard deviation divided by the square root of the sample size from the determined mean value.

### 3.2 Assessment of protease inhibitors as inhibitors of OATP1B1 in vitro

**FIGURE 2:**
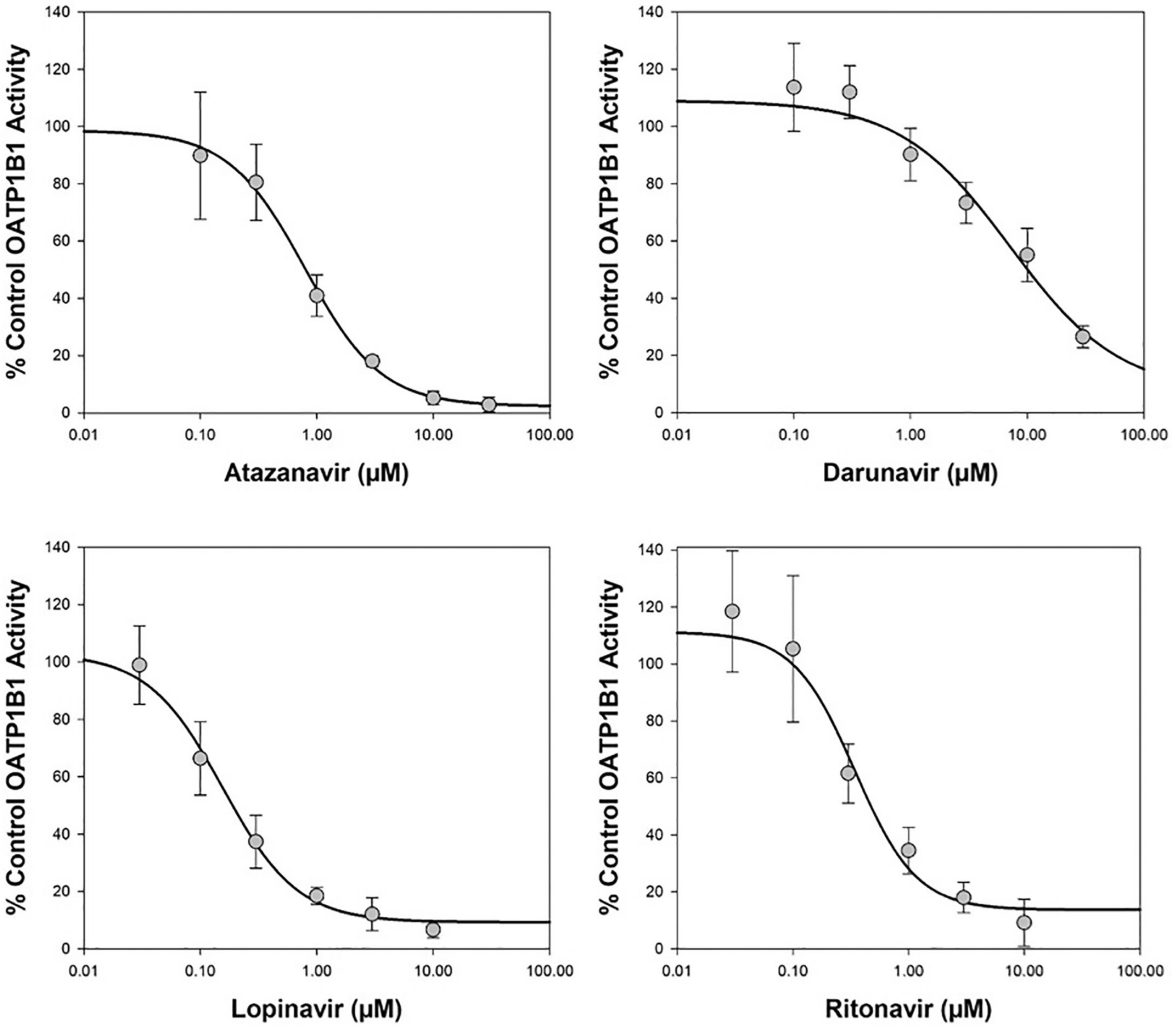
Representative plot of mean concentration‐dependent inhibition of OATP1B1‐mediated transport of [^3^H]‐estradiol 17β‐D‐glucuronide (0.02 μM) by atazanavir, darunavir, lopinavir or ritonavir. Data are expressed as mean (± standard deviation) from 9 wells per incubation condition (triplicate wells on three separate occasions).

Atazanavir, darunavir, lopinavir or ritonavir demonstrated reproducible inter‐assay concentration‐dependent inhibition of OATP1B1‐mediated transport of [^3^H]‐estradiol 17β‐D‐glucuronide (0.02 μM) with a determined mean (±SD) IC_50_ value of 0.734 ± 0.177 μM (Table 1 and Figure 2), 9.53 ± 2.50 μM (Table 2 and Figure 2), 0.220 ± 0.0655 μM (Table 3 and Figure 2), or 0.497 ± 0.125 μM (Table 4 and Figure 2), respectively. Assessment of DDI potential via inhibition of hepatic OATP1B1 using the current FDA ^23^ static equation approach gave R = 1 + [I_in max u_]/K_i_ values of 7.68, 1.13, 3.05 or 1.03, respectively, indicating the potential for interaction in vivo (≥1.1; see Table 5 for [I] value) perpetrated by atazanavir, darunavir and lopinavir, but not by ritonavir. The positive control inhibitor, rifamycin SV (100 μM) gave acceptable inhibition (mean of 96.5 ± 3.2%) in the test system.

**TABLE 5:** Pharmacokinetic parameters of protease inhibitors in clinical interaction studies with rosuvastatin.

Perpetrator drug	Dose (mg)	MW	[I_g_] (μM)	[C_max_] (μM)	f_u_	[I_in max_] (μM)	[I_in max u_] (μM)
Atazanavir	300	704.90	142	8.68^a^	0.14	35.0	4.90
Darunavir	600	547.73	73.0^b^	10.6^c^	0.05	24.2^b^	1.21
Lopinavir	400	628.80	42.4^d^	14.6^e^	0.02	22.5^d^	0.450
Ritonavir	100	720.95	1.39^f^	0.930^a^	0.011	1.19^f^	0.0131

*Note*: f_u_ = fraction unbound (taken from the drug label accessed via Drugs@FDA database; www.accessdata.fda.gov/scripts/cder/daf). [I_g_] = maximal enterocyte concentration and [I_in max_] = maximum hepatic inlet concentration; calculated as described in Materials and Methods. [C_max_] = Mean steady‐state maximum plasma concentration for total (bound plus unbound) drug.

^a^ Value taken from Elsby et al.^1^

^b^ Value for k_a_ taken to be 0.02.^35^

^c^ Value taken from Kakuda et al.^36^

^d^ Value for k_a_ taken to be 0.02.^37^

^e^ Value taken from Elsby et al.^13^

^f^ Value for k_a_ taken to be 0.003.^16^

### 3.3 Assessment of protease inhibitors as inhibitors of OATP1B3, NTCP and OAT3 in vitro

**FIGURE 3:**
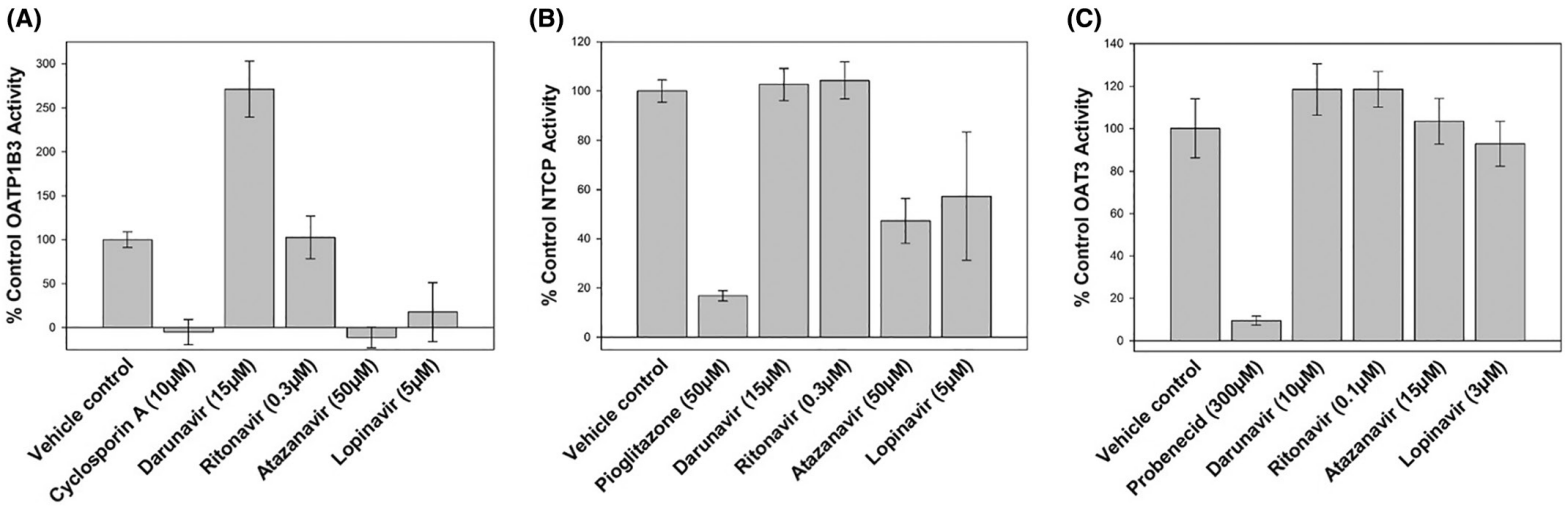
Representative plot of mean single concentration inhibition of (A) OATP1B3‐mediated transport of [^3^H]‐estradiol 17β‐D‐glucuronide (0.02 μM), (B) NTCP‐mediated transport of [^3^H]‐taurocholic acid (1 μM), and C) OAT3‐mediated transport of [^3^H]‐estrone 3‐sulfate (1 μM) by darunavir, ritonavir, atazanavir or lopinavir and respective positive control inhibitors. Data are expressed as mean (± standard deviation) from 6 wells per incubation condition (triplicate wells on two separate occasions).

Single concentration inhibition screens conducted on two separate occasions determined that none of the protease inhibitors caused inhibition of OAT3‐mediated [^3^H]‐estrone 3‐sulfate (1 μM) transport when tested at concentrations greater than or equal to ten times their unbound [C_max_] (Figure 3), thereby indicating no DDI potential via renal OAT3. The positive control inhibitor, probenecid (300 μM) gave acceptable inhibition (mean of 90.6%) in the test system. Furthermore, neither darunavir nor ritonavir when tested at a single concentration greater than or equal to ten times their [I_in max u_] produced inhibition of either OATP1B3‐ or NTCP‐mediated probe substrate transport (Figure 3), indicating no DDI potential of the two drugs via those hepatic transporters. The OATP1B3 and NTCP positive control inhibitors, cyclosporin A (10 μM) and pioglitazone (50 μM), respectively, gave acceptable inhibition (mean of 100.0% and 83.2 %) in their test systems.

**FIGURE 4:**
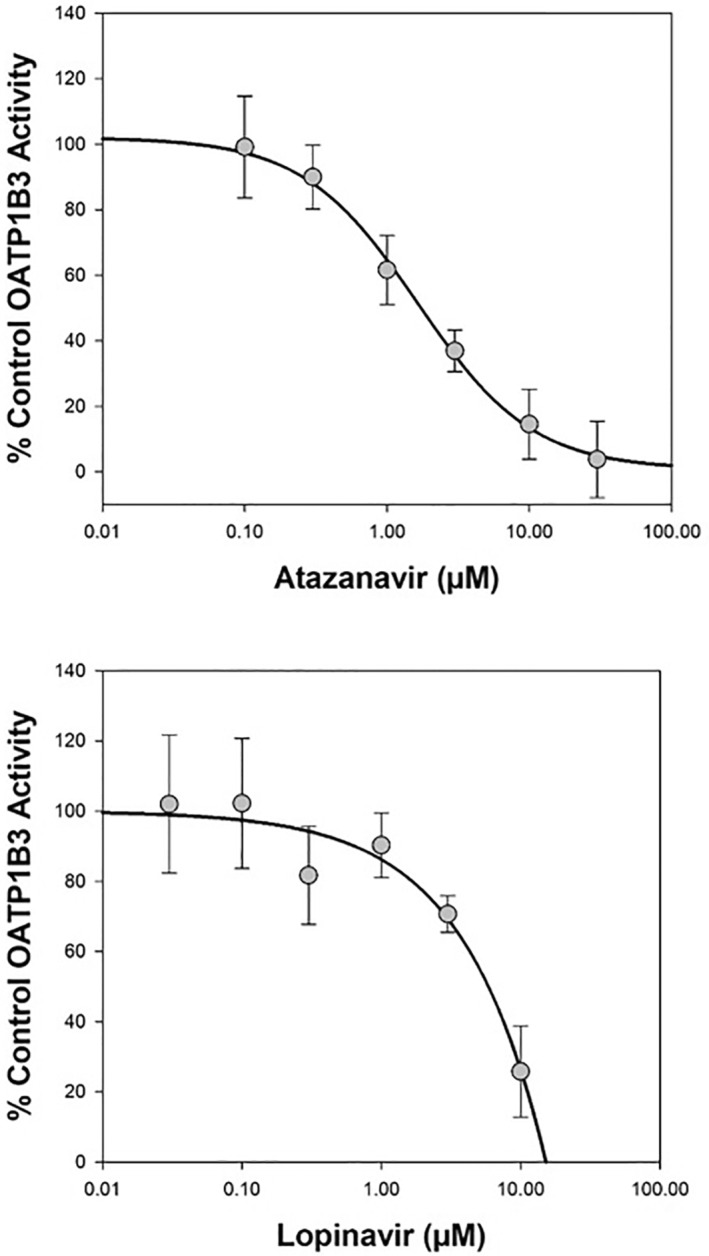
Representative plot of mean concentration‐dependent inhibition of OATP1B3‐mediated transport of [^3^H]‐estradiol 17β‐D‐glucuronide (0.02 μM) by atazanavir or lopinavir. Data are expressed as mean (± standard deviation) from 9 wells per incubation condition (triplicate wells on three separate occasions).

**FIGURE 5:**
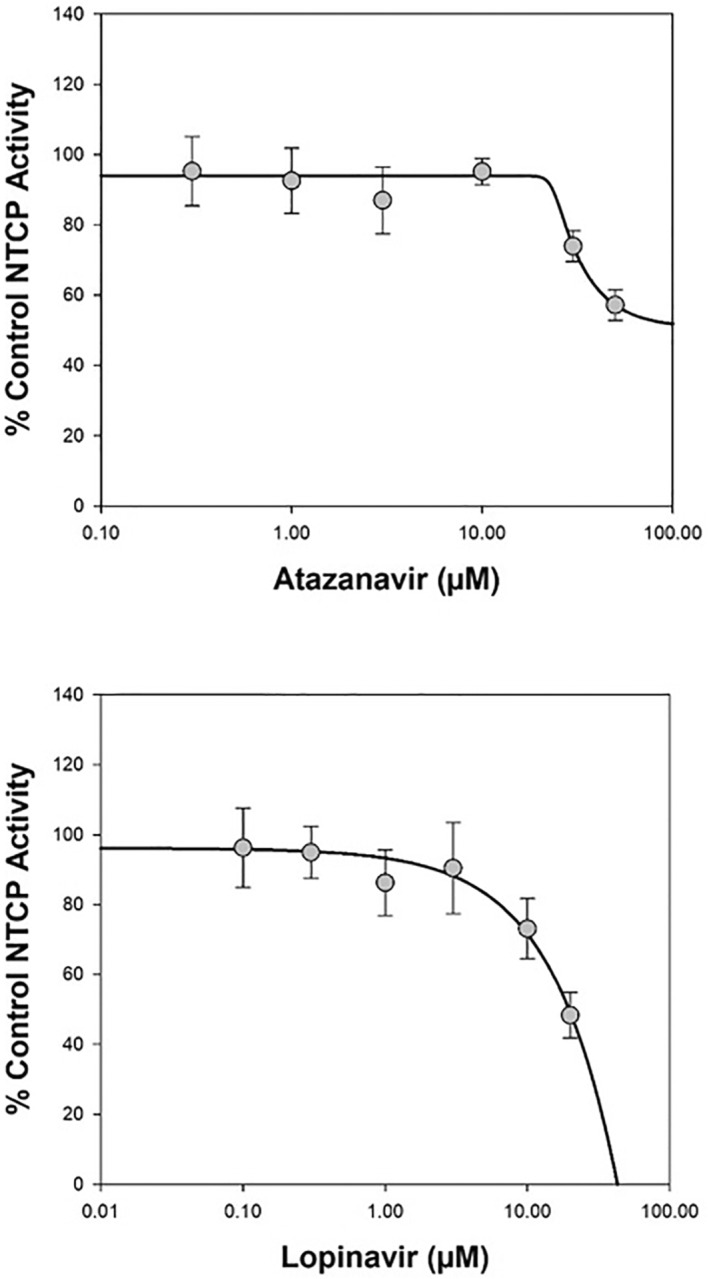
Representative plot of mean concentration‐dependent inhibition of NTCP‐mediated transport of [^3^H]‐taurocholic acid (1 μM) by atazanavir or lopinavir. Data are expressed as mean (± standard deviation) from 9 wells per incubation condition (triplicate wells on three separate occasions).

Both atazanavir and lopinavir produced inhibition of OATP1B3 and NTCP vehicle control transport activity in the screens (Figure 3) leading to subsequent determination of their IC_50_ value at each transporter. Atazanavir demonstrated reproducible inter‐assay concentration‐dependent inhibition of OATP1B3‐mediated transport of [^3^H]‐estradiol 17β‐D‐glucuronide (0.02 μM), or NTCP‐mediated transport of [^3^H]‐taurocholic acid (1 μM), with mean (±SD) IC_50_ values of 1.86 ± 0.500 μM, or 65.6 ± 10.7 μM, respectively (Table 1 and Figures 4 and 5). This gave a corresponding R value of 3.63 or 1.07, respectively, indicating the potential for interaction in vivo (≥1.1) via inhibition of OATP1B3 but not via NTCP for atazanavir. The concentration‐dependent inhibition of OATP1B3 and NTCP by lopinavir gave mean (±SD) IC_50_ values of 5.04 ± 0.0950 μM (R = 1.09) and 20.3 ± 2.13 μM (R = 1.02), respectively (Table 3 and Figs. 4 and 5), indicating no DDI potential of the two drugs via those hepatic transporters. Cyclosporin A (mean inhibition of 95.8 ± 6.6%) and pioglitazone (mean inhibition of 80.4 ± 5.1%) gave acceptable positive control inhibition in their respective test systems.

### 3.4 Predicted versus observed rosuvastatin AUC changes with protease inhibitors based upon in vitro transporter inhibitory data

Using the mean drug inhibitory parameters determined above for atazanavir, darunavir, lopinavir and ritonavir versus BCRP, OATP1B1, OATP1B3 or NTCP and their pharmacokinetic parameters provided in Table 5, calculations were performed with mechanistic static equations (1 and 2) in order to predict the theoretical fold‐increase in rosuvastatin AUC that would occur following co‐administration. Calculated AUC ratios (AUCR) due to inhibition of each transporter alone, and in combination, are shown in Table 6. The theoretical AUCR due to inhibition of BCRP alone ranged from 1.03 to 1.63 and was higher than that attributed to each drug's corresponding inhibition of OATP1B1 (1.01 to 1.49). The overall predicted AUCR due to inhibition of both intestinal and hepatic transporters was within 1.09‐, 1.18‐ and 1.03‐fold of the clinical AUCR observed during DDI with atazanavir, darunavir and lopinavir, respectively.

**TABLE 6:** Predicted versus observed AUC increases of rosuvastatin following co‐administration with atazanavir, darunavir, lopinavir or ritonavir based upon inhibition of intestinal BCRP (*ƒ*_e_ = 0.5) and inhibition of hepatic OATP1B1 (*ƒ*_e_ = 0.38), OATP1B3 (*ƒ*_e_ = 0.11) or NTCP (*ƒ*_e_ = 0.21) or cumulative inhibition of all hepatic uptake transport (*ƒ*_e_ = 0.7).

Perpetrator drug	Dose (mg)	Predicted fold increase in AUC due to inhibition of composite pathways					Overall predicted AUCR	Clinically observed AUCR
		Intestinal BCRP (theoretical max = 2.0)	OATP1B1 (theoretical max = 1.61)	OATP1B3 (theoretical max = 1.12)	NTCP (theoretical max = 1.27)	All hepatic uptake (theoretical max = 3.33)		
Atazanavir	300	**1.63** [1.55–1.73]	1.49 [1.44–1.56]	1.09 [1.07–1.11]	1.01 [1.01–1.02]	**1.74** [1.63–1.92]	**2.84** [2.53–3.32]	3.10
Darunavir	600	1.20 [1.17–1.25]	1.04 [1.03–1.11]	N.A.	N.A.	‐	**1.25** [1.21–1.39]	1.48 [1.0‐2.1]#
Lopinavir	400	**1.58** [1.49–1.71]	1.34 [1.26–1.51]	1.01 [1.01–1.01]	1.00 [1.00–1.01]	**1.37** [1.28–1.55]	**2.16** [1.91–2.65]	2.10 [1.7‐2.6]#
Ritonavir	400	1.03 [1.02–1.06]	1.01 [1.01–1.03]	N.A.	N.A.	‐	**1.04** [1.03–1.09]	N.R.

*Note*: N.A.—no inhibition was observed at an incubation concentration ≥ 10x [I] for the transporter interaction site, indicating DDI via this mechanism as unlikely. N.R.—no clinical AUCR reported for sole administration of ritonavir. [ ] numbers in square brackets are the calculated predicted AUCR range using the upper and lower 95% confidence intervals determined for each inhibitory kinetic parameter given in Tables 1, 2, 3, 4. To determine the lower and upper predicted AUCR, the upper and lower confidence interval for K_i_ values were used for calculations, respectively. [ ]# numbers in square brackets are the calculated 90% confidence interval stated on the Crestor® drug label. Where applicable, the individual numbers in bold highlight the major pathway(s) implicated in the DDI to give the overall predicted AUCR number.

## 4 DISCUSSION

Statins are common comedications across many disease indications and elevated plasma concentrations are linked to increased risk of myopathy.^24^ For this reason, it is beneficial for project teams to be able to contextualize the clinical risk an investigational drug might perpetrate with statins by effectively predicting and quantifying DDI.^16^, ^25^ Quantitative DDI prediction can be performed through use of either the less resource intensive mechanistic static equation approach or through a physiologically based pharmacokinetic (PBPK) dynamic approach. Whilst the former could theoretically overestimate DDI if too conservative input parameters are used as assumptions, the same can equally be said of the latter dynamic approach, which is in fact a build of the mechanistic model from the perspective of the perpetrator drug, coupled with system parameters and potentially more assumptions around a required larger number of input parameters. It is important to remember that the majority of oral drug pharmacokinetic transporter‐mediated DDIs are driven primarily by first pass effects on absorption and hepatic elimination (rather than by systemic plasma trough concentrations). This is the reason why mechanistic static equation models predict effectively well despite employing theoretical maximum concentrations of the perpetrator drug at the interaction site, as time‐related parameters have less impact on such DDIs. Indeed, the use of mechanistic static equations for accurately predicting DDI has been applied successfully to 28 clinically observed statin DDIs with an accuracy of 97%, thereby proving the effectiveness of the approach for quantitative DDI prediction.^1^, ^13^, ^16^, ^19^

When considering the victim rosuvastatin, a mechanistic static model incorporating inhibition of critical disposition pathways intestinal BCRP and hepatic OATP1B1, correctly predicted 91% of clinical DDIs.^13^, ^16^ However, the DDI perpetrated by atazanavir underpredicted the clinical situation when focusing only on these pathways and appeared to reconcile the observed AUCR when inhibition of all hepatic uptake (*ƒ*_e_ = 0.7, vs *ƒ*_e OATP1B1_ = 0.38) was also considered. For rosuvastatin, the critical pathways that culminate in its hepatic elimination in order of importance (based on contribution; defined by *ƒ*_e_) are OATP1B1, NTCP and OATP1B3. Consequently, it was necessary to assess atazanavir as an inhibitor of BCRP, OATP1B1, NTCP, OATP1B3 and OAT3 (involved in renal elimination) to establish whether these additional pathways need to be incorporated into the mechanistic model to more accurately predict observed AUCR, aiding confirmation of the mechanism underpinning the DDI. To understand whether a similar mechanistic scenario might exist for other rosuvastatin‐protease inhibitor DDIs, darunavir, lopinavir and ritonavir's inhibitory properties were also assessed and used in predictions for confirming their mechanisms of DDI.

In an ideal world, assessment of inhibition potential would utilize the victim drug of the DDI under investigation (e.g., rosuvastatin) as the in vitro probe substrate in order to determine the clinically relevant IC_50_ value for prediction of risk. However, the reality is that this is often not the case for practical reasons (e.g., the lack of commercial availability of drug substance), but predominantly because the pharmaceutical industry (through regulatory guidance) generally favors the use of a single established in vitro “surrogate” probe substrate to evaluate routine inhibitory liability against specific transporters and metabolizing enzymes that can be subsequently applied to holistically risk assess perpetrators against multiple victim drugs. With respect to each specific transporter, in line with regulatory recommendations (since inhibition can be substrate‐dependent), the chosen in vitro probe substrate should either be clinically relevant to observed DDIs, a good surrogate of a clinically relevant in vivo substrate, or one that generates a lower IC_50_ for known inhibitors to avoid underestimating interaction potential.^16^, ^23^ The chosen in vitro transporter probe substrates utilized in this study all fall under one of the latter two recommendations. Indeed, it is well established in the literature that estradiol 17β‐D‐glucuronide is a good and recommended surrogate in vitro OATP1B1 probe substrate for clinically relevant OATP1B1 substrate drugs (e.g., statins) that can be used to avoid substrate‐dependent IC_50_ variation when assessing inhibition potential.^26^, ^27^ To further prevent any possible substrate‐dependent differences in inhibition potential in the present study for extrapolating to rosuvastatin DDIs, for all the individual transporters studied, the probe substrate concentration incubated was at least ten‐times lower than its determined K_m_ value at the transporter so that the calculated IC_50_ values equated to K_i_ values. Consequently, as such inhibition constants are widely accepted to be independent of substrate, then this ensures the right inhibitory parameter value is being determined for accurate DDI prediction. In addition, inclusion of a pre‐incubation step with a range of concentrations of inhibitor (for all transporters) acts to ensure that any adsorption of inhibitor to plasticware during the assay (were it to occur) is mitigated due to the masking of non‐specific binding sites prior to performing the co‐incubation with fresh inhibitor solution and probe substrate. Therefore, the final concentrations of inhibitor would be anticipated to remain as nominal further ensuring the accuracy of the determined K_i_ value.^16^

For assessment of inhibition of BCRP function, the present study has utilized the regulatory industry gold standard methodology involving the polarized Caco‐2 cell monolayer test system, which has previously been extensively validated to correctly identify inhibitors of BCRP using rosuvastatin as probe substrate.^13^ In this study, the prototypical BCRP substrate estrone 3‐sulfate was used as a good surrogate in vitro probe substrate for rosuvastatin since established BCRP inhibitors gave similar K_i_ values to those obtained with rosuvastatin.^16^ The reason why estrone 3‐sulfate is a good surrogate is due to the similar hydrophilic properties it shares with rosuvastatin (existing predominantly in the charged anionic form at physiological pH) resulting in its subsequent mechanistic translatability to rosuvastatin vectorial (basolateral to apical) transport processes in Caco‐2 cells. Due to their negligible intrinsic passive membrane permeability, both estrone 3‐sulfate and rosuvastatin require an active basolateral uptake process in order to enter cells to interact with apically expressed BCRP (for which they are selective substrates). In Caco‐2 cells this uptake is mediated by the passive facilitative organic solute transporter alpha/beta (OSTα/β^28^, ^29^). Indeed, it is because of this shared mechanistic property that employing an alternative polarized cell system overexpressing BCRP (i.e., MDCK‐BCRP) is actually detrimental to the use of clinically relevant rosuvastatin, or its surrogate estrone 3‐sulfate, as probe substrate for BCRP function due to the fact that MDCK‐BCRP cells are deficient in the basolateral uptake component, thereby hindering their entry into cells. In contrast to Caco‐2, this deficiency in MDCK‐BCRP precludes its use as a test system for the correct identification of highly polar substrates or inhibitors of BCRP.^29^ This also explains why MDCK‐BCRP assays require the use of more permeable/lipophilic BCRP probe substrates (e.g., cladribine, prazosin) in order to observe good transport function which, unfortunately, are not relatable to hydrophilic rosuvastatin as the major victim of clinically significant BCRP‐mediated DDIs.^16^ Regarding the Caco‐2 test system, the similarity and ranking of K_i_ values for the known BCRP inhibitors novobiocin, Ko143, cyclosporin A, pantoprazole, sulfasalazine, atorvastatin, diclofenac, fluvastatin and nifedipine obtained in the previous validation with corresponding literature values derived from inside‐out membrane vesicles expressing BCRP,^13^ gives confidence that the mechanism of inhibition observed in the Caco‐2 test system reflects perturbation of BCRP efflux rather than of basolateral uptake and, as such, the resulting determined K_i_ is the correct one for assessing DDI risk at BCRP.

The present study confirmed that all the protease inhibitor drugs investigated inhibited BCRP and OATP1B1 with the same rank order of inhibitory potency of lopinavir > ritonavir > atazanavir >> darunavir at both transporters. The determined mean BCRP IC_50_ (K_i_) values were within 1.3‐ to 2‐fold of published literature values for atazanavir, darunavir, lopinavir or ritonavir (Tables 1, 2, 3, 4),^13^, ^15^, ^30^ confirming the suitability of estrone 3‐sulfate as a good surrogate in vitro probe substrate for rosuvastatin.^16^ Furthermore, the determined mean OATP1B1 IC_50_ (K_i_) values were also within 1.2‐ to 2‐fold of previous studies that utilized either the same recommended surrogate OATP1B1 in vitro probe substrate estradiol 17β‐D‐glucuronide^26^, ^27^ or the clinically relevant probe atorvastatin, for atazanavir, darunavir or lopinavir (Tables 1, 2, 3).^13^, ^31^

For investigations involving OAT3, NTCP and OATP1B3, initial inhibition experiments were performed using a single inhibitor concentration screening format in which each of the protease inhibitor drugs were assessed at a concentration that was ≥10‐times either their systemic unbound maximum plasma concentration (for renal OAT3) or their unbound maximum hepatic inlet concentration (for hepatic NTCP and OATP1B3). These drug‐specific concentrations were chosen so that in the absence of any observed inhibition in vitro, based on regulatory basic static equation cut‐offs ([I_max u_]/K_i_ ≥0.1 or R=1+[I_in max u_]/K_i_ ≥1.1),^23^ the drug could be confirmed as not having the potential to cause DDI through inhibition of that respective transporter in vivo. The results from the present study indicate that neither atazanavir, darunavir, lopinavir nor ritonavir inhibited OAT3 transport which is consistent with the findings of Yoshida et al.^32^ who, with the exception of ritonavir, also showed no inhibition up to 10 μM. Whilst an IC_50_ (4.7 μM) was reported for ritonavir, this value is 47‐times higher than the clinically relevant concentration (0.1 μM) tested in this study. This confirms that OAT3 perturbation is not involved as a mechanism in the observed DDIs between rosuvastatin and these protease inhibitors. With respect to NTCP and OATP1B3, neither darunavir nor ritonavir inhibited these transporters at clinically relevant hepatic concentrations confirming that these pathways are also not involved in the mechanism of their respective rosuvastatin DDIs. Conversely, both atazanavir and lopinavir inhibited OATP1B3 and NTCP in the current study. The determined mean OATP1B3 IC_50_ (K_i_) value for atazanavir was 3.9‐fold lower than previously published versus atorvastatin as probe substrate.^33^ This likely reflects the more accurate determination of inhibition in this study due to the presence of a pre‐incubation step with test inhibitor which was absent in the earlier published study performed prior to inclusion of such recommendations in regulatory guidance (draft DDI guidance).^34^ This is because if atazanavir's inhibitory mechanism toward OATP1B1 involves *trans*‐inhibition alongside *cis*‐inhibition, then the 2014 study would only reflect *cis*‐inhibition, whereas the inclusion of the pre‐incubation step in this study would also account for any additional effect of *trans*‐inhibition on determined IC_50_ value. This same explanation likely explains the disconnect between the present study and that of Vildhede et al.^33^ who showed no inhibition of NTCP by atazanavir (tested up to 40 μM) and no inhibition of NTCP or OATP1B3 by lopinavir (tested up to 10 μM).

Using mechanistic modeling, the predicted rosuvastatin AUCR due to combined inhibition of BCRP and OATP1B1 by atazanavir still fell short of the clinically observed value (2.43 vs 3.1), despite the fold increase due to OATP1B1 (1.49) being close to its theoretical maximum (1.6‐fold) and indicating that there is around 80% inhibition of the transporter. This is likely the result of not accounting for the modest effect on AUCR of inhibition of OATP1B3 and NTCP by atazanavir. Whilst small, the predicted AUC increase of 1.09‐fold due to OATP1B3 inhibition actually represents 75% inhibition of this transporter's function (based on the theoretical maximum of 1.12‐fold), whereas the predicted AUCR due to NTCP inhibition (1.01) only reflects a 4% reduction in function. For these hepatic transporters, the magnitude of inhibition by atazanavir directly correlates with the calculated R‐value. To calculate a predicted AUCR due to combined inhibition of OATP1B1, OATP1B3 and NTCP by atazanavir it is not appropriate mathematically to simply multiply the individual AUCR values together because conceptually they are not isolated clearance pathways, rather they contribute to the overall hepatic elimination pathway of rosuvastatin. Therefore, it is this combined *ƒ*_e_ value and the sum of each transporters respective [I]/K_i_ ratio (Equation 2) that is used in the mechanistic equation. Despite the predicted minimal effect on rosuvastatin AUCR from individual inhibition of OATP1B3 and NTCP by atazanavir, the cumulative effect of these pathways with inhibition of OATP1B1 gave a predicted increase in AUC that was 25% higher than inhibition of OATP1B1 alone. Furthermore, when this combined all hepatic AUCR was multiplied by the AUCR due to inhibition of BCRP then the overall predicted AUCR (2.84; 2.53–3.32) for the rosuvastatin‐atazanavir DDI reconciled with the clinically observed AUCR (3.1), confirming the underlying mechanism in DDI to be inhibition of BCRP, OATP1B1, OATP1B3 and NTCP.

Whilst a similar effect with regard to inhibition of OATP1B3 and NTCP was observed with lopinavir, the individual predicted AUCR values are minimal at 1.01 and 1.00, respectively, which is consistent with the R‐values being below threshold. Nevertheless, despite the suggested lack of interaction, inhibition of both transporters still contributed to overall DDI via all hepatic uptake, as evidenced by the small increase in cumulative AUCR compared with that from OATP1B1 alone. When this cumulative prediction was combined with that due to BCRP, the overall predicted AUCR (2.16) matched the clinical AUCR. However, predictions based solely on BCRP and OATP1B1 (AUCR = 2.12) also match the clinical observation, suggesting that for lopinavir, the driving mechanism of DDI is simply inhibition of intestinal BCRP and OATP1B1 as previously reported.^13^

In the case of darunavir, since it is not an inhibitor of OATP1B3 or NTCP, the mechanism for perpetrating its DDI is principally inhibition of BCRP and, to a lesser extent, inhibition of OATP1B1. The AUCR predictions in this study are marginally lower than that of Elsby et al.^13^ and likely reflect the 2‐fold higher BCRP K_i_ determined here (reducing predictions from 1.33 to 1.20). Finally, whilst ritonavir is a potent inhibitor of both BCRP and OATP1B1 in vitro, it only flags for the potential to cause DDI in vivo through inhibition of intestinal BCRP, whereas due to a combination of low dose and low unbound exposure, it is not predicted to cause DDI through OATP1B1. The DDI risk due to BCRP inhibition is diminished further when enterocyte, rather than theoretical lumen (used in guidance), concentration is considered in the mechanistic model, thereby resulting in a predicted AUCR of only 1.03. When combined, the overall AUCR is predicted to be 1.04 indicating that ritonavir is unlikely to cause a clinical DDI with rosuvastatin if administered in isolation. In clinical practice however, ritonavir is coadministered to boost the absorption of a second protease inhibitor through inhibition of intestinal enzymes.

In conclusion, determined transporter inhibitory parameters and use of mechanistic static model predictions have successfully indicated that the clinical DDIs perpetrated by atazanavir, darunavir and lopinavir with rosuvastatin are driven by inhibition of intestinal BCRP and hepatic OATP1B1 with minimal contribution from OATP1B3, NTCP and OAT3. Furthermore, the use of mechanistic models for quantitative DDI prediction may help illuminate the involvement of additional pathways in DDI where predictions do not reconcile the clinical observation.

## AUTHOR CONTRIBUTIONS

Participated in research design: Atkinson, Coghlan, Edgerton, Elsby, Hodgson, Outteridge. Conducted experiments: Coghlan, Edgerton, Hodgson. Performed data analysis: Atkinson, Coghlan, Edgerton, Elsby, Hodgson, Outteridge. Wrote or contributed to the writing of the manuscript: Atkinson, Coghlan, Edgerton, Elsby, Hodgson, Outteridge.

## CONFLICT OF INTEREST STATEMENT

The authors are all employees, or a former employee (HC), of Cyprotex Discovery Ltd (an Evotec Company) which is a contract research organization.

## Data Availability

The authors confirm that the data that support the findings of this study are available from the corresponding author upon reasonable request.
